# Supplementary material for: Competing-risks model for predicting the prognostic value of lymph nodes in medullary thyroid carcinoma
Source: PLoS One. 2023 Oct 16;18(10):e0292488. doi: 10.1371/journal.pone.0292488 (PMC10578593; doi:10.1371/journal.pone.0292488)
Supplement: S1 Table — (DOCX) [file pone.0292488.s001.docx]

Table 1 Patients characteristics and demographics(DSS)

| Variables | Total (n = 2435) | Survival (n = 2237) | Death(n = 198) | p |
| --- | --- | --- | --- | --- |
| Age, Median (Q1, Q3) | 54 (42, 65) | 53 (42, 65) | 60 (48, 70.75) | < 0.001 |
| Sex, n (%) |  |  |  | < 0.001 |
| Female | 1439 (59.1) | 1355 (60.57) | 84 (42.42) |  |
| Male | 996 (40.9) | 882 (39.43) | 114 (57.58) |  |
| Race, n (%) |  |  |  | 0.113 |
| Black | 185 (7.6) | 171 (7.64) | 14 (7.07) |  |
| Others | 173 (7.1) | 166 (7.42) | 7 (3.54) |  |
| White | 2077 (85.3) | 1900 (84.94) | 177 (89.39) |  |
| Marital, n (%) |  |  |  | 0.211 |
| Divorced/Separated | 162 (6.65) | 147 (6.57) | 15 (7.58) |  |
| Married | 1499 (61.56) | 1366 (61.06) | 133 (67.17) |  |
| Single/Unmarried | 462 (18.97) | 434 (19.4) | 28 (14.14) |  |
| Widowed/Others | 312 (12.81) | 290 (12.96) | 22 (11.11) |  |
| Diagnosis, n (%) | |  |  | < 0.001 |
| 1998~2000 | 193 (7.93) | 158 (7.06) | 35 (17.68) |  |
| 2001~2005 | 499 (20.49) | 419 (18.73) | 80 (40.4) |  |
| 2006~2010 | 723 (29.69) | 674 (30.13) | 49 (24.75) |  |
| 2011~2016 | 1020 (41.89) | 986 (44.08) | 34 (17.17) |  |
| AJCC, n (%) |  |  |  | < 0.001 |
| I~III | 672 (27.6) | 666 (29.77) | 6 (3.03) |  |
| IV | 303 (12.44) | 266 (11.89) | 37 (18.69) |  |
| Unknown | 1460 (59.96) | 1305 (58.34) | 155 (78.28) |  |
| AJCC.T, n (%) |  |  |  | < 0.001 |
| T0~T2 | 719 (29.53) | 705 (31.52) | 14 (7.07) |  |
| T3~T4 | 275 (11.29) | 246 (11) | 29 (14.65) |  |
| Tx/Unknown | 1441 (59.18) | 1286 (57.49) | 155 (78.28) |  |
| AJCC.N, n (%) |  |  |  | < 0.001 |
| N0 | 570 (23.41) | 564 (25.21) | 6 (3.03) |  |
| N1 | 429 (17.62) | 392 (17.52) | 37 (18.69) |  |
| Nx/Unknown | 1436 (58.97) | 1281 (57.26) | 155 (78.28) |  |
| AJCC.M, n (%) |  |  |  | < 0.001 |
| NO | 960 (39.43) | 930 (41.57) | 30 (15.15) |  |
| Unknown | 1427 (58.6) | 1272 (56.86) | 155 (78.28) |  |
| YES | 48 (1.97) | 35 (1.56) | 13 (6.57) |  |
| Hist.type, n (%) |  |  |  | < 0.001 |
| MTC | 2117 (86.94) | 1970 (88.06) | 147 (74.24) |  |
| MTCAS | 318 (13.06) | 267 (11.94) | 51 (25.76) |  |
| Radiotherapy, n (%) | |  |  | < 0.001 |
| NO/Unknown | 2099 (86.2) | 1980 (88.51) | 119 (60.1) |  |
| YES | 336 (13.8) | 257 (11.49) | 79 (39.9) |  |
| Radiation.recode, n (%) | |  |  | < 0.001 |
| EBRT | 229 (9.4) | 160 (7.15) | 69 (34.85) |  |
| IRT | 103 (4.23) | 94 (4.2) | 9 (4.55) |  |
| Others | 2103 (86.37) | 1983 (88.65) | 120 (60.61) |  |
| Chemotherapy, n (%) | |  |  | < 0.001 |
| NO | 2370 (97.33) | 2197 (98.21) | 173 (87.37) |  |
| YES | 65 (2.67) | 40 (1.79) | 25 (12.63) |  |
| Tumor.size, n (%) | |  |  | < 0.001 |
| <=2cm | 910 (37.37) | 890 (39.79) | 20 (10.1) |  |
| 2~4cm | 543 (22.3) | 505 (22.57) | 38 (19.19) |  |
| Size>4cm | 323 (13.26) | 276 (12.34) | 47 (23.74) |  |
| Unknown | 659 (27.06) | 566 (25.3) | 93 (46.97) |  |
| Sequence.number2, n (%) | |  |  | < 0.001 |
| 1 | 2099 (86.2) | 1901 (84.98) | 198 (100) |  |
| 2~ | 336 (13.8) | 336 (15.02) | 0 (0) |  |
| Number.1, n (%) | |  |  | < 0.001 |
| 1 | 1837 (75.44) | 1660 (74.21) | 177 (89.39) |  |
| 2~ | 598 (24.56) | 577 (25.79) | 21 (10.61) |  |
| Grade, n (%) |  |  |  | < 0.001 |
| I~II | 142 (5.83) | 130 (5.81) | 12 (6.06) |  |
| III~IV | 83 (3.41) | 60 (2.68) | 23 (11.62) |  |
| Unknown | 2210 (90.76) | 2047 (91.51) | 163 (82.32) |  |
| Survival. months, Median (Q1, Q3) | 67 (28, 116) | 69 (30, 120) | 37 (17, 82.75) | < 0.001 |
| Outcome, n (%) |  |  |  | < 0.001 |
| 0 | 1986 (81.56) | 1986 (88.78) | 0 (0) |  |
| 1 | 449 (18.44) | 251 (11.22) | 198 (100) |  |
| Outcome3, n (%) | |  |  | < 0.001 |
| 0 | 1986 (81.56) | 1986 (88.78) | 0 (0) |  |
| 1 | 198 (8.13) | 0 (0) | 198 (100) |  |
| 2 | 251 (10.31) | 251 (11.22) | 0 (0) |  |
| Proportion, Median (Q1, Q3) | 0 (0, 0.27) | 0 (0, 0.22) | 0.45 (0.09, 0.77) | < 0.001 |
| Age.cat, n (%) |  |  |  | < 0.001 |
| ~45 | 750 (30.8) | 707 (31.6) | 43 (21.72) |  |
| 46~60 | 824 (33.84) | 763 (34.11) | 61 (30.81) |  |
| 61~ | 861 (35.36) | 767 (34.29) | 94 (47.47) |  |
| LNR, n (%) |  |  |  | < 0.001 |
| ~25% | 1774 (72.85) | 1701 (76.04) | 73 (36.87) |  |
| 25%~53% | 332 (13.63) | 292 (13.05) | 40 (20.2) |  |
| 54%~ | 329 (13.51) | 244 (10.91) | 85 (42.93) |  |
| PLNs, n (%) |  |  |  | < 0.001 |
| 0 | 1446 (59.38) | 1401 (62.63) | 45 (22.73) |  |
| 1~10 | 640 (26.28) | 561 (25.08) | 79 (39.9) |  |
| 11~75 | 349 (14.33) | 275 (12.29) | 74 (37.37) |  |
| Total_LNs, n (%) |  |  |  | < 0.001 |
| 0~2 | 873 (35.85) | 820 (36.66) | 53 (26.77) |  |
| 19~89 | 783 (32.16) | 687 (30.71) | 96 (48.48) |  |
| 3~18 | 779 (31.99) | 730 (32.63) | 49 (24.75) |  |

Abbreviations: EBRT, external beam radiotherapy; IRT, iodine radiotherapy; Sequence.number2, history of previous malignant tumors; Number.1.2~, number of lesions; Outcome0, survival; Outcome1, Disease-specific death; Outcome2, Other deaths.
