# Supplementary figures and images for: Competing-risks model for predicting the prognostic value of lymph nodes in medullary thyroid carcinoma
Source: PLoS One. 2023 Oct 16;18(10):e0292488. doi: 10.1371/journal.pone.0292488 (PMC10578593; doi:10.1371/journal.pone.0292488)

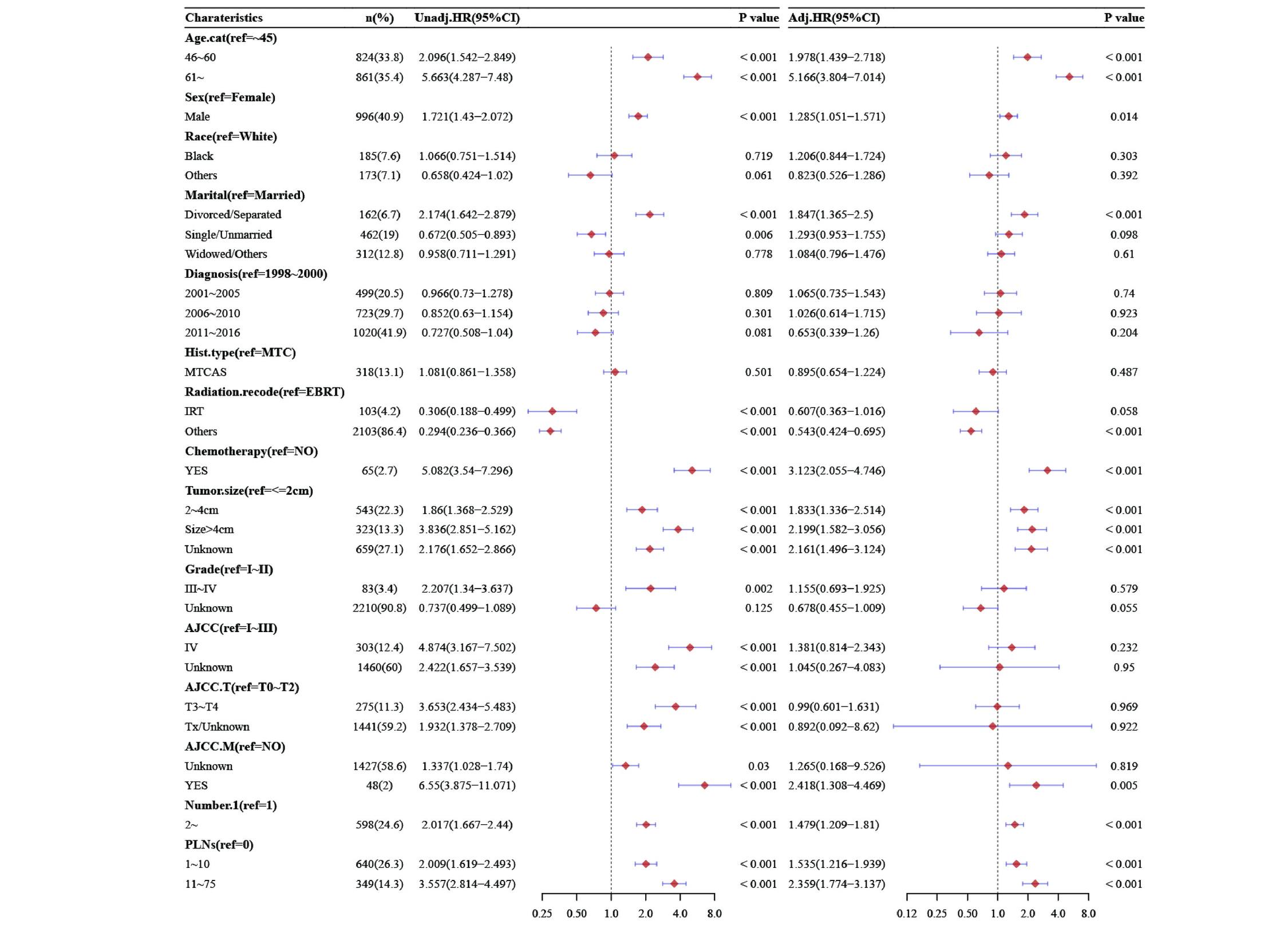

Supplement: S1 Fig — (TIF) [file pone.0292488.s002.tif]

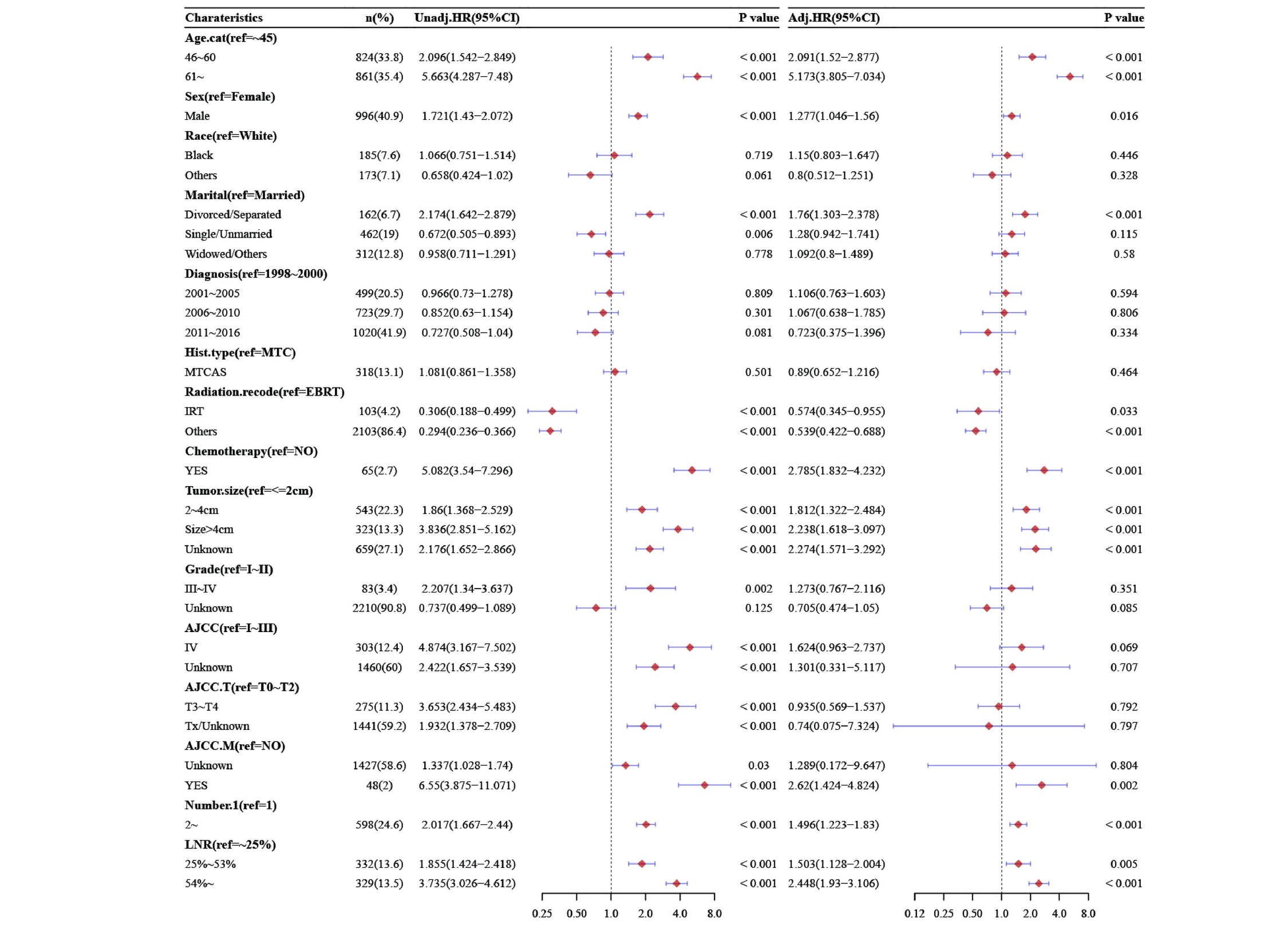

Supplement: S2 Fig — (TIF) [file pone.0292488.s003.tif]

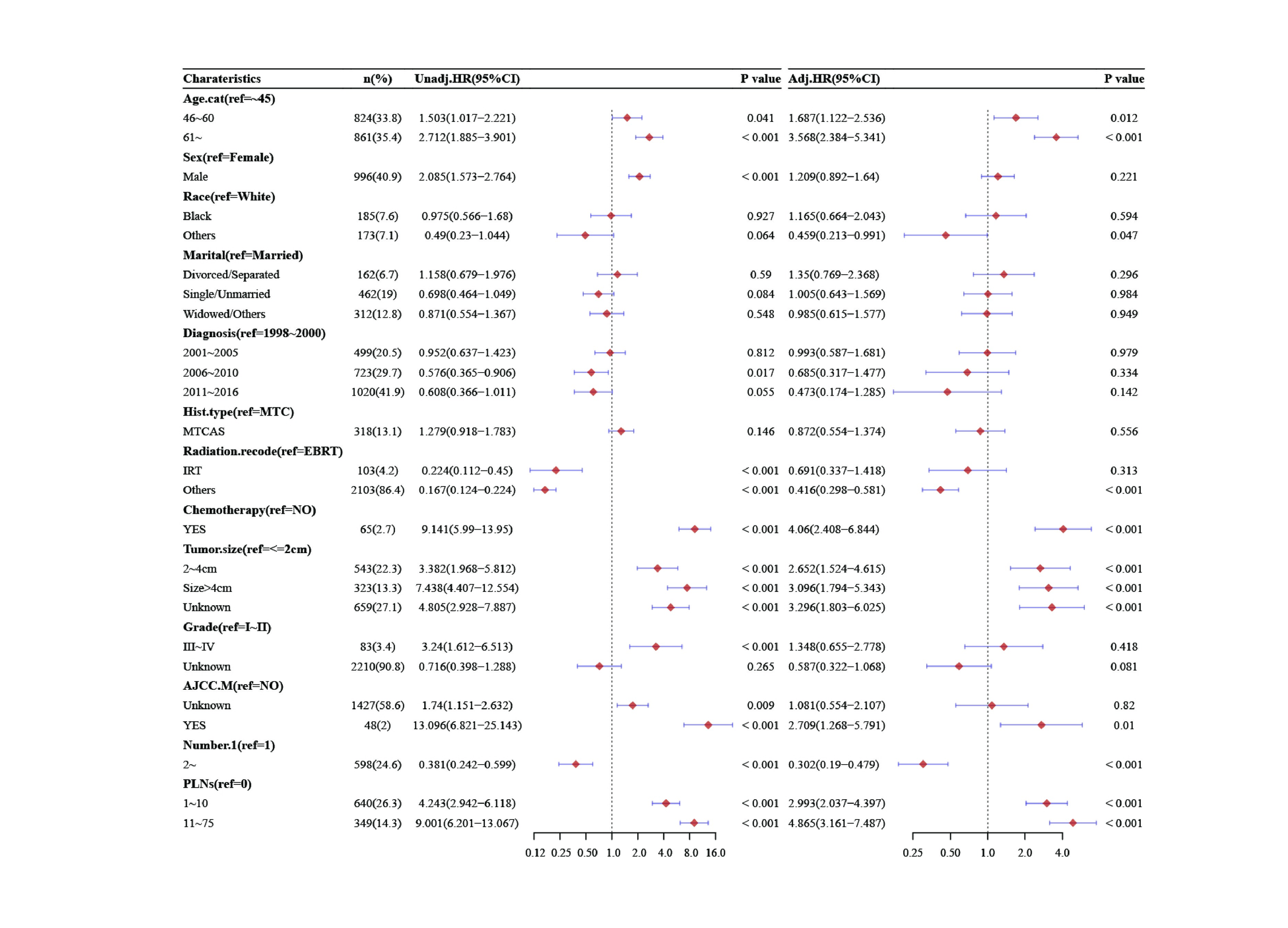

Supplement: S3 Fig — (TIF) [file pone.0292488.s004.tif]

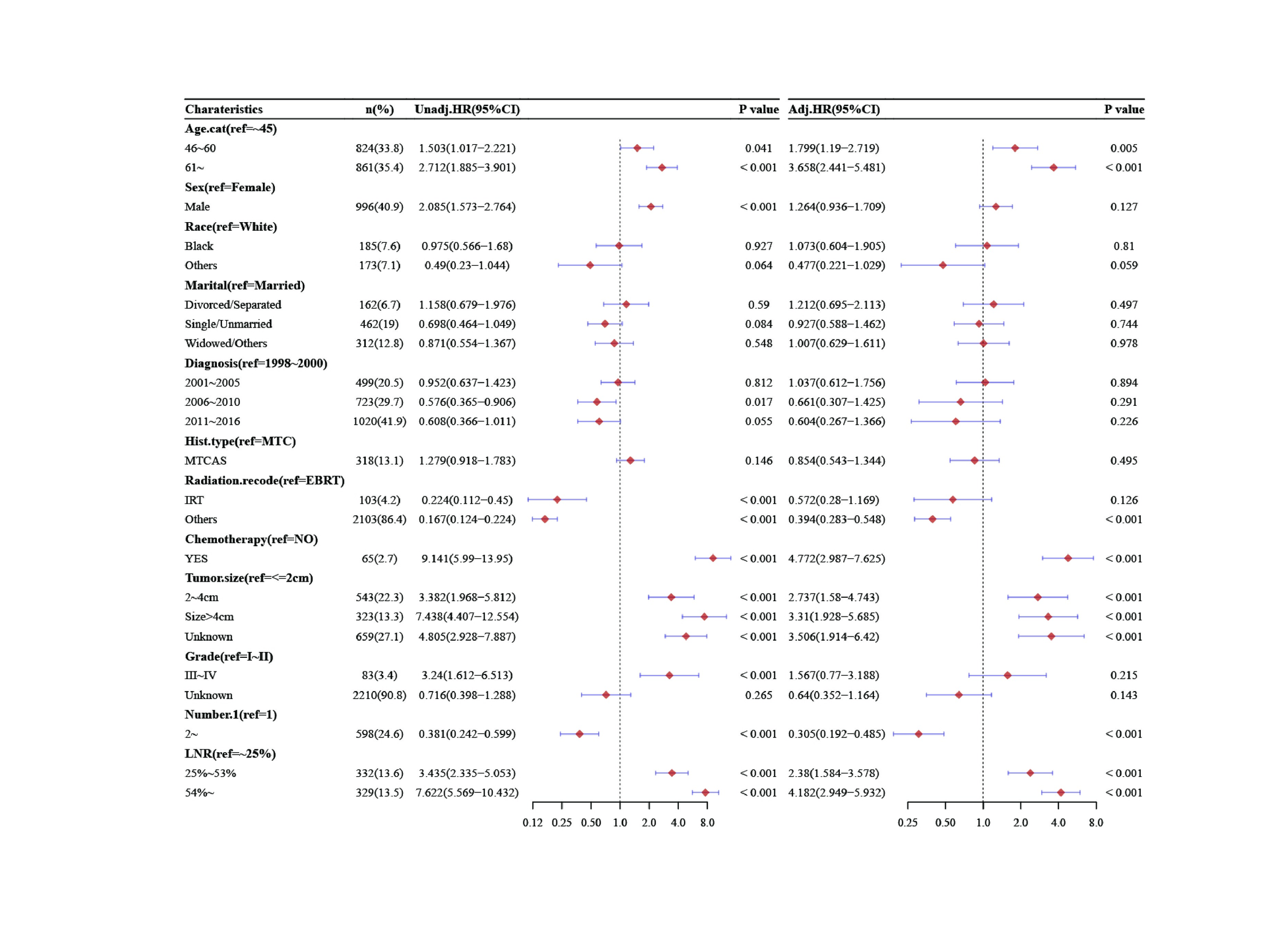

Supplement: S4 Fig — (TIF) [file pone.0292488.s005.tif]
