# Supplementary material for: Competing-risks model for predicting the prognostic value of lymph nodes in medullary thyroid carcinoma
Source: PLoS One. 2023 Oct 16;18(10):e0292488. doi: 10.1371/journal.pone.0292488 (PMC10578593; doi:10.1371/journal.pone.0292488)
Supplement: Attachment — Submitted filename: Response to Reviewers.docx [file pone.0292488.s006.docx]

Dear Editor Antonino Maniaci and Reviewers, thanks very much for taking your time to review this manuscript. I really appreciate all your comments and suggestions! Please find my itemized responses in below and my revisions in the re-submitted files.

Thanks again!

SUGGESTIONS FROM EDITOR

1.Please ensure that your manuscript meets PLOS ONE's style requirements, including those for file naming.

2. PLOS requires an ORCID iD for the corresponding author in Editorial Manager on papers submitted after December 6th, 2016.

Thank you for your feedback. I have revised the manuscript format as required and improved the ORCID information.

COMMENTS TO THE AUTHOR:

Reviewer #1:

Major
1.Comment: The manuscript must be edited for English.

1.Reply: We apologize for the poor language of our manuscript. We worked on the manuscript for a long time and the repeated addition and removal of sentences and sections obviously led to poor readability. We have now worked on both language and readability and have also involved native English speakers for language corrections. We really hope that the flow and language level have been substantially improved.

2.Comment: It is not clear what OS and DSS mean, as it is not well specified in the paper. This makes the paper very hard to read and understand.

2.Reply: The definitions of OS and DSS are written in the last paragraph of 2.1 Data Collection and Patient Selection in this article, and I have added the corresponding complete spelling to help understand.

Minor
1.Comment: Please specify in the abstract and in the methods that this was a retrospective study.

1.Reply: Sorry for neglecting this point. I have already annotated the retrospective study in the abstract and methods.

2.Comment: Sometimes the word lymph node is abbreviated, sometimes it is not, thus confounding the reader.

2.Reply: I have corrected my writing to make the paper easy to understand.

Reviewer #2:

1.Comment: Provide a systematic review of the relevant literature to establish the need for this study. Discuss the limitations of previous studies that this one aimed to address.

1.Reply: Thank you for pointing out the shortcomings of the article, and I have added relevant explanations in the discussion section.

2.Comment: Describe your study design more clearly in the Methods section. Include details like:
Inclusion and exclusion criteria
Data collection procedures
How variables were classified and coded
Statistical analyses, including packages used
Report measures of uncertainty for effect estimates in the Results, e.g. 95% confidence intervals.

2.Reply: I have added corresponding content in the materials and methods section and made adjustments to make it clearer.

3.Comment: Discuss limitations more thoroughly, e.g.:
Potential selection bias in the SEER database
Limited information on treatments
Retrospective study design
Possible residual confounding
Limited generalizability due to predominantly white sample

3.Reply: I have supplemented the limitations of the research at the end of the discussion section.

Thanks again for the constructive feedback.
