# Supplementary material for: Competing-risks model for predicting the prognostic value of lymph nodes in medullary thyroid carcinoma
Source: PLoS One. 2023 Oct 16;18(10):e0292488. doi: 10.1371/journal.pone.0292488 (PMC10578593; doi:10.1371/journal.pone.0292488)
Supplement: Attachment — Submitted filename: Response to Reviewers.docx [file pone.0292488.s007.docx]

Dear Editor Antonino Maniaci and Reviewers, thanks very much for taking your time to review this manuscript. I really appreciate all your comments and suggestions! Please find my itemized responses in below and my revisions in the re-submitted files.

Thanks again!

COMMENTS TO THE AUTHOR:

Reviewer #2:

1.Comment: Provide a systematic review of the relevant literature to establish the need for this study. Discuss the limitations of previous studies that this one aimed to address.

1.Reply: Thank you for pointing out the shortcomings of the article. I have addressed them by supplementing the discussion section with a literature review and limitations of previous studies. I have also included relevant information from the American Thyroid Association guidelines and the literature on lymph node dissection in medullary thyroid cancer, as you mentioned. I apologize for not being able to locate the original article on the prognostic value of competing-risks models compared with Cox regression for breast cancer patients, but I have reviewed several similar articles and added a comprehensive summary in the discussion section.

2.Comment: Describe your study design more clearly in the Methods section. Include details like:
Inclusion and exclusion criteria
Data collection procedures
How variables were classified and coded
Statistical analyses, including packages used
Report measures of uncertainty for effect estimates in the Results, e.g. 95% confidence intervals.

2.Reply: In the previous revision, I have added corresponding content in the materials and methods section and made adjustments to make it clearer.

3.Comment: Discuss limitations more thoroughly, e.g.:
Potential selection bias in the SEER database
Limited information on treatments
Retrospective study design
Possible residual confounding
Limited generalizability due to predominantly white sample

3.Reply: In the previous revision, I have supplemented the limitations of the research at the end of the discussion section.

4. Provide more nuanced conclusions that qualify your findings and highlight implications for future research and clinical practice.
Consult subject experts and/or epidemiologists on your statistical methods, results and conclusions.
Cite relevant literature throughout to contextualize your study and support claims.
Edit the manuscript for clarity, conciseness and adherence to reporting guidelines for observational studies (e.g. STROBE).
Consider registering the study protocol to improve transparency and reduce reporting bias.

4.Thank you for your feedback. I have added some content to the discussion section of the article to make it more organized and clear, with detailed results and accurate arguments.

Thanks again for the constructive feedback.
